# A Rare Case of Methicillin-Susceptible Staphylococcus aureus Catheter-Related Bloodstream Infection Complicated by Acute Bacterial Meningitis and Refractory Septic Shock in Morocco: A Case Report

**DOI:** 10.7759/cureus.108432

**Published:** 2026-05-07

**Authors:** Youssef Benoumrhar, Nada Khlifi Tagzouti, Youssef El Kamouni, Said Zouhair, Lamiae Arsalane

**Affiliations:** 1 Department of Microbiology, Avicenna Military Hospital, Marrakech, MAR; 2 Faculty of Medicine and Pharmacy, Cadi Ayyad University, Marrakech, MAR

**Keywords:** catheter-related bloodstream infection, differential time to positivity, hematogenous meningitis, septic shock, staphylococcus aureus bacteremia

## Abstract

Catheter-related bloodstream infections (CRBSIs) remain a serious complication in patients with hematologic malignancies, regardless of the causative microorganism, although *Staphylococcus aureus* remains an important pathogen because of its potential for rapid progression and metastatic complications. This report details the case of a 58-year-old man receiving immunochemotherapy for diffuse large B-cell lymphoma who developed fulminant sepsis 72 hours following the implantation of a totally implantable venous access port. The patient presented with high-grade fever, acute confusion, and local signs of port-site infection. Blood cultures confirmed methicillin-susceptible *S. aureus* bacteremia consistent with a catheter-related source. The diagnosis of secondary acute bacterial meningitis was confirmed by cerebrospinal fluid analysis and culture. Despite prompt initiation of empirical antimicrobial therapy, de-escalation to targeted anti-staphylococcal therapy, and catheter removal, the patient’s condition rapidly deteriorated, and the patient died of refractory septic shock. This case highlights the aggressive progression and high mortality associated with *S. aureus* CRBSI complicated by hematogenous meningitis in immunocompromised patients and illustrates the importance of early recognition, prompt microbiological diagnosis, immediate source control, and vigilant clinical monitoring following catheter placement.

## Introduction

Central venous catheters are essential for ensuring reliable vascular access for chemotherapy, transfusions, and supportive care in patients with hematologic malignancies. Despite advancements in insertion techniques and preventive strategies, catheter-related bloodstream infections (CRBSIs), defined as bloodstream infections arising from colonization or infection of an intravascular catheter, remain a significant source of morbidity and mortality among hematology and oncology patients [1,2]. Immunosuppression from malignancy and chemotherapy-induced cytopenias increases the risk of severe infections and poor outcomes. [1,3].

Among the pathogens responsible for CRBSIs, *Staphylococcus aureus* warrants particular attention due to its virulence, its ability to adhere to intravascular devices promoting biofilm formation, and its strong propensity for hematogenous dissemination, that is, spread through the bloodstream to distant sites [4]. Although coagulase-negative staphylococci are more frequently isolated from catheter-related infections, *S. aureus* is more commonly associated with higher complication rates, including metastatic infections and septic shock, particularly in immunocompromised hosts [4,5].

Acute bacterial meningitis is among the most severe but rare complications of *S. aureus* bacteremia. Although rare, *S. aureus* meningitis has been reported to account for approximately 1%-9% of bacterial meningitis cases [6]. In a large multicenter adult cohort of 350 cases of *S. aureus* meningitis, methicillin-susceptible strains represented 232 cases, corresponding to 66% of cases, indicating that MSSA remains epidemiologically important in this clinical entity [6]. Hematogenous *S. aureus* meningitis is particularly rare; in a nationwide Danish study, it was identified in 96 of 12,480 episodes of *S. aureus* bacteremia/sepsis, corresponding to 0.8%, and was associated with a mortality rate of 56% [7]. In adults, outside the neurosurgical or post-traumatic context, *S. aureus* meningitis typically reflects hematogenous spread from a distant focus such as infective endocarditis, spondylodiscitis, or an infected vascular access device [8]. Despite modern antimicrobial therapy and intensive care management, the mortality rate associated with *S. aureus* meningitis remains high [8,9].

We present a case of fulminant methicillin-susceptible *S. aureus* (MSSA) CRBSI occurring shortly after implantation of a totally implantable venous access port in a patient with diffuse large B-cell lymphoma, complicated by acute bacterial meningitis and refractory septic shock. This case is notable for the very early onset after port placement, the microbiological evidence supporting a catheter-related source, and the rapid progression from localized port-site infection to hematogenous meningitis and fatal septic shock despite prompt management.

## Case presentation

A 58-year-old man, known to have diffuse large B-cell lymphoma diagnosed in February 2026 after presenting with an abdominal mass that had been progressively evolving for six months and staged as IVB according to the Ann Arbor classification, presented electively for insertion of a totally implantable venous access port, with no other acute complaint at admission. He was on treatment with R-CHOP chemotherapy (cyclophosphamide, doxorubicin, vincristine, and prednisone) in combination with rituximab and had received one cycle before admission. His past medical history was otherwise unremarkable, with no documented history of diabetes mellitus, chronic kidney disease, previous bloodstream infection, or prior catheter-related infection. At admission for port implantation, the patient was alert and oriented, with a baseline Glasgow Coma Scale score of 15/15, no neck stiffness, and no focal neurological deficit. Baseline laboratory tests conducted upon admission for implantation of a totally implantable venous access port revealed hypochromic microcytic anemia and significant lymphopenia.

The totally implantable venous access port was inserted on the day of admission under standard aseptic conditions. No immediate peri-procedural complications, including bleeding, hematoma, pneumothorax, catheter malposition, or respiratory instability, were documented. Seventy-two hours post-port implantation, the patient developed high-grade fever (39.2°C) associated with tachycardia and progressive neurological deterioration, including confusion and decreased level of consciousness. The Glasgow Coma Scale score upon clinical examination was 13/15, with eye opening to verbal stimulus, confused verbal response, and obeying motor commands (E3V4M6). The patient was initially hemodynamically and respiratorily stable. Local examination of the port site revealed erythema and edema, suggestive of an early insertion-site infection.

Given suspected sepsis with neurological impairment, an urgent diagnostic workup was initiated. Four sets of blood cultures were collected, including two sets drawn from the implantable port and two sets from peripheral veins at different anatomical sites. Paired central and peripheral blood cultures were obtained simultaneously with identical blood volumes to facilitate accurate assessment of differential time to positivity (DTP), in accordance with recommended diagnostic strategies for CRBSI. Further hematologic and biochemical investigations were performed. Serial laboratory results demonstrated marked biological deterioration, with pronounced elevation of inflammatory markers followed by progressive pancytopenia (Table 1). This worsening paralleled the patient’s neurological progression, characterized by persistent confusion and decreased level of consciousness, and was subsequently followed by hemodynamic deterioration, ultimately progressing to refractory septic shock. Overall, the clinical course was characterized by three key warning features: very early onset of fever after port implantation, local inflammatory signs at the port site, and rapidly progressive neurological impairment followed by hemodynamic deterioration.

**Table 1:** Serial hematologic, inflammatory, and biochemical parameters from baseline port placement to 24 hours after symptom onset Serial laboratory monitoring showed a marked inflammatory response, with rising C-reactive protein and procalcitonin levels, followed by progressive pancytopenia. These biological changes paralleled the patient’s neurological worsening and subsequent hemodynamic deterioration.

Parameter	Baseline (day 0; port placement)	Symptom onset (72 hours after admission)	24 hours after symptom onset	Reference range
White blood cell count (cells/µL)	9,590	2,670	260	4,000-11,000
Neutrophils (cells/µL)	9,450	2,540	180	1,400-7,700
Lymphocytes (cells/µL)	60	70	60	1,000-4,800
Monocytes (cells/µL)	70	10	10	180-1,000
Eosinophils (cells/µL)	0	0	0	20-630
Basophils (cells/µL)	10	50	10	0-110
Hemoglobin (g/dL)	9.2	8.8	8.7	13.0-18.0
Hematocrit (%)	26.9	25.9	25	40-52
Mean corpuscular volume (fL)	76.6	76.9	75.1	78-98
Mean corpuscular hemoglobin (pg)	26.2	26.1	26.1	26-34
Mean corpuscular hemoglobin concentration (g/dL)	34.2	34	34.8	31-36
Platelet count (×10³/µL)	227	185	32	150-450
Blood urea nitrogen (g/L)	0.25	0.32	0.51	0.15-0.45
Serum creatinine (mg/L)	4.38	5.40	7.74	6.8-13.6
C-reactive protein (mg/L)	146.8	218.9	428.7	<5
Procalcitonin (ng/mL)	0.4	3.2	6.5	<0.5
Uric acid (µmol/L)	107.6	118	129	210-420
Lactate dehydrogenase (U/L)	240	259	444	135-225

Following the exclusion of contraindications via a brain computed tomography (CT) scan, a lumbar puncture was performed. Macroscopically, the cerebrospinal fluid (CSF) was clear. CSF analysis showed a leukocyte count of 512 cells/mm³ with a pronounced neutrophilic predominance (99%), hypoglycorrhachia (CSF glucose 1.56 mmol/L), and hyperproteinorrachia (190 mg/dL). CSF Gram stain was negative. CSF culture was performed using the three-point inoculation technique on solid media (blood agar, chocolate agar, and mannitol salt agar), followed by aerobic incubation at 37°C for 24 hours. Empirical antimicrobial therapy was promptly initiated with intravenous vancomycin (20 mg/kg every 12 hours) and ceftriaxone at meningitis dosing (2 g every 12 hours). Renal function remained normal, and no dose adjustment was required.

A chest radiograph was performed, revealing no pulmonary abnormalities. Transthoracic echocardiography showed no valvular vegetations.

**Figure 1:**
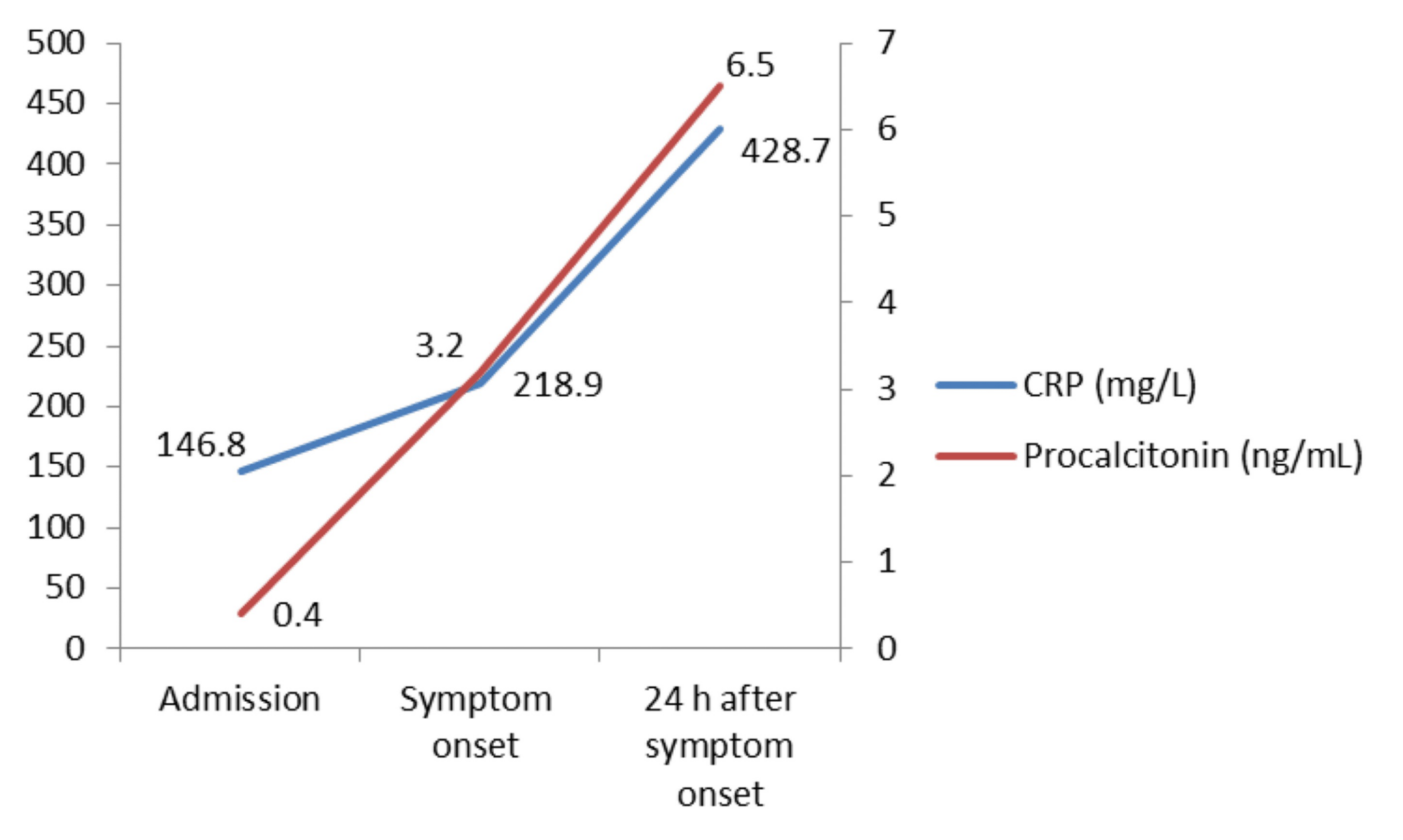
Temporal evolution of C-reactive protein and procalcitonin from admission to 24 hours after symptom onset C-reactive protein (CRP) is plotted on the left y-axis (mg/L), and procalcitonin is plotted on the right y-axis (ng/mL).

Laboratory follow-up showed a progressive inflammatory response. At admission, C-reactive protein (CRP) was already elevated at 146.8 mg/L, while procalcitonin was within the reference range (0.4 ng/mL). At symptom onset (72 hours after admission), CRP further increased to 218.9 mg/L, and procalcitonin increased significantly to 3.2 ng/mL. Within the following 24 hours, coinciding with clinical deterioration, CRP reached 428.7 mg/L and procalcitonin rose to 6.5 ng/mL, supporting the diagnosis of severe bacterial sepsis and paralleling the patient’s neurological and hemodynamic worsening (Figure 1).

**Figure 2:**
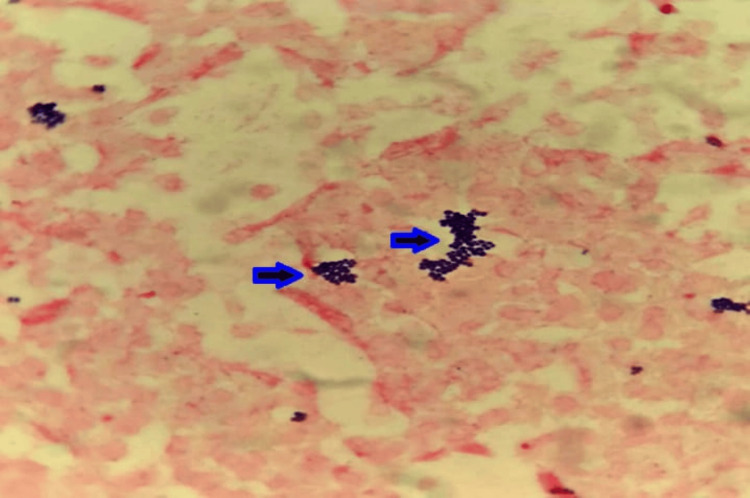
Gram stain (×1,000, oil immersion) showing numerous Gram-positive cocci in grape-like clusters

**Figure 3:**
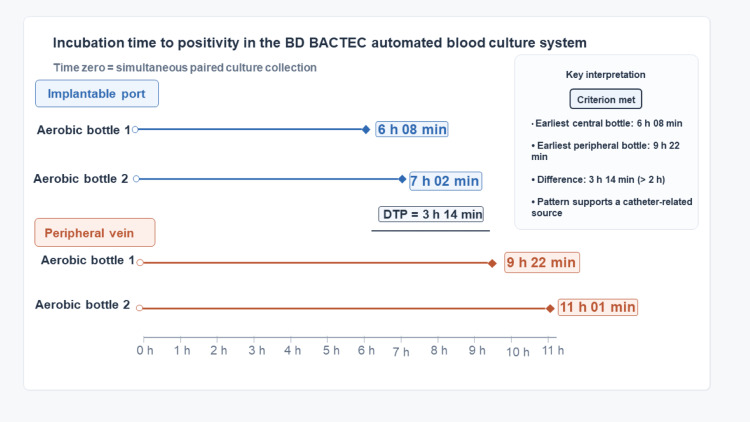
Differential time to positivity in paired central and peripheral blood cultures Earlier positivity of central blood culture bottles than peripheral bottles, with a differential time to positivity greater than two hours. This finding supported the totally implantable venous access port as the likely source of bloodstream infection.

The first aerobic blood culture bottle drawn from the implantable venous access port became positive after six hours and eight minutes of incubation in the BD BACTEC automated blood culture system (Becton, Dickinson and Company, Franklin Lakes, NJ). The second central aerobic bottle became positive after seven hours and two minutes. Gram stain showed Gram-positive cocci in clusters (Figure 2). Peripheral aerobic blood culture bottles became positive after nine hours and 22 minutes and 11 hours and one minute of incubation, respectively. Compared with the earliest positive central bottle, this corresponded to delays of three hours and 14 minutes and four hours and 53 minutes, fulfilling the DTP criterion (>2 hours) suggestive of CRBSI (Figure 3). This earlier positivity of central blood cultures supported the implantable venous access port as the likely source of bacteremia and prompted consideration of early source control.

**Figure 4:**
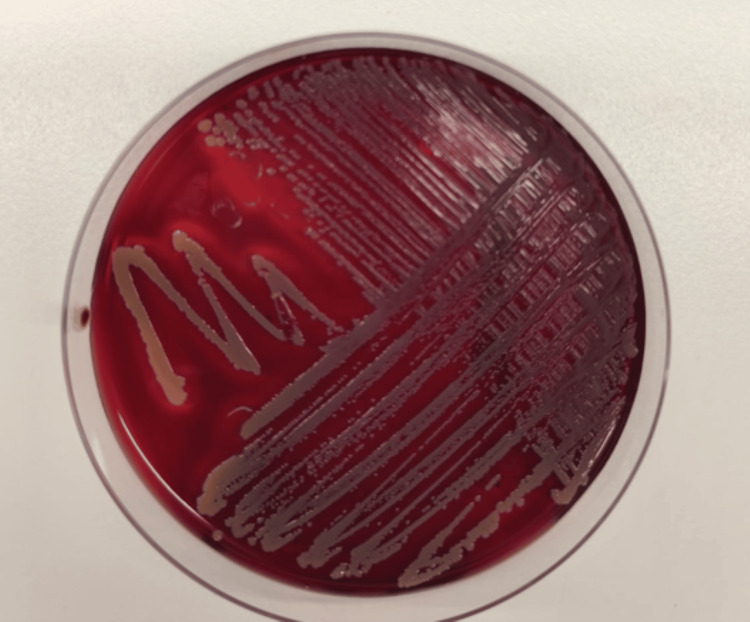
Colony morphology of Staphylococcus aureus on blood agar after 24 hours of incubation After 24 hours of aerobic incubation at 37°C, colonies appeared smooth, convex, and opaque, with a creamy texture and a surrounding zone of β-hemolysis on blood agar. This colony morphology was consistent with *S. aureus* and supported the presumptive microbiological identification before definitive confirmation.

Subculture from positive blood cultures was performed on blood agar, chocolate agar, and mannitol salt agar, and incubated aerobically at 37°C. After 24 hours of incubation, colonies were smooth, convex, and opaque with a creamy texture and characteristic golden-yellow pigmentation with a surrounding zone of β-hemolysis on blood agar (Figure 4). Microscopic examination confirmed Gram-positive cocci arranged in clusters. Biochemical testing demonstrated catalase and coagulase positivity.

To guide early antimicrobial decision-making, antimicrobial susceptibility testing was first performed by disk diffusion on Mueller-Hinton agar (John Howard Mueller and Jane Hinton, Harvard University, Cambridge, MA) from the positive blood culture bottle. Definitive species identification and antimicrobial susceptibility tests were subsequently performed using the BD Phoenix™ M50 automated system (Becton, Dickinson and Company, Franklin Lakes, NJ) (Table 2), and results were interpreted according to the Antibiogram Committee of the French Society for Microbiology/European Committee on Antimicrobial Susceptibility Testing (CA-SFM/EUCAST) 2025 guidelines [10]. The isolate was identified as MSSA with a Phoenix confidence level of 99%.

**Table 2:** Antimicrobial susceptibility profile of the methicillin-susceptible Staphylococcus aureus isolate determined by the BD Phoenix™ M50 system and interpreted according to CA-SFM/EUCAST 2025 criteria Minimum inhibitory concentrations (MICs) are reported in mg/L. Interpretations follow CA-SFM/EUCAST 2025 breakpoints; “I” denotes susceptible, increased exposure according to the EUCAST definition. Cefoxitin was used as a screening marker for methicillin susceptibility. The susceptibility profile confirmed an MSSA phenotype and supported de-escalation to targeted anti-staphylococcal therapy. CA-SFM/EUCAST, Antibiogram Committee of the French Society for Microbiology/European Committee on Antimicrobial Susceptibility Testing; MSSA, methicillin-susceptible *Staphylococcus aureus*

Antibiotic	MIC (mg/L)	Interpretation (CA-SFM/EUCAST 2025)
Gentamicin	≤1	Susceptible (S)
Cefoxitin	≤2	Susceptible (S)
Penicillin G	>0.5	Resistant (R)
Oxacillin	0.5	Susceptible (S)
Amoxicillin-clavulanate	≤2/1	Susceptible (S)
Daptomycin	≤1	Susceptible (S)
Trimethoprim-sulfamethoxazole	≤2/38	Susceptible (S)
Teicoplanin	≤1	Susceptible (S)
Vancomycin	≤1	Susceptible (S)
Clindamycin	≤0.25	Susceptible (S)
Erythromycin	≤0.25	Susceptible (S)
Fusidic acid	≤1	Susceptible (S)
Linezolid	≤2	Susceptible (S)
Ciprofloxacin	≤1	Susceptible, increased exposure (I)
Levofloxacin	≤1	Susceptible, increased exposure (I)
Moxifloxacin	≤0.25	Susceptible (S)
Rifampin	≤0.25	Susceptible (S)
Tetracycline	≤0.5	Susceptible (S)

On the following day, CSF culture returned positive, yielding the same MSSA strain with a comparable antimicrobial susceptibility profile. These concordant microbiological findings established an MSSA CRBSI complicated by hematogenous meningitis. Based on the microbiological documentation, antimicrobial therapy was de-escalated to high-dose intravenous cloxacillin at meningitis dosing (2 g every four hours). Thus, the main results guiding clinical management were the earlier positivity of central blood cultures supporting a catheter-related source, confirmation of MSSA in blood and CSF cultures, and the susceptibility profile allowing de-escalation to targeted anti-staphylococcal therapy.

**Figure 5:**
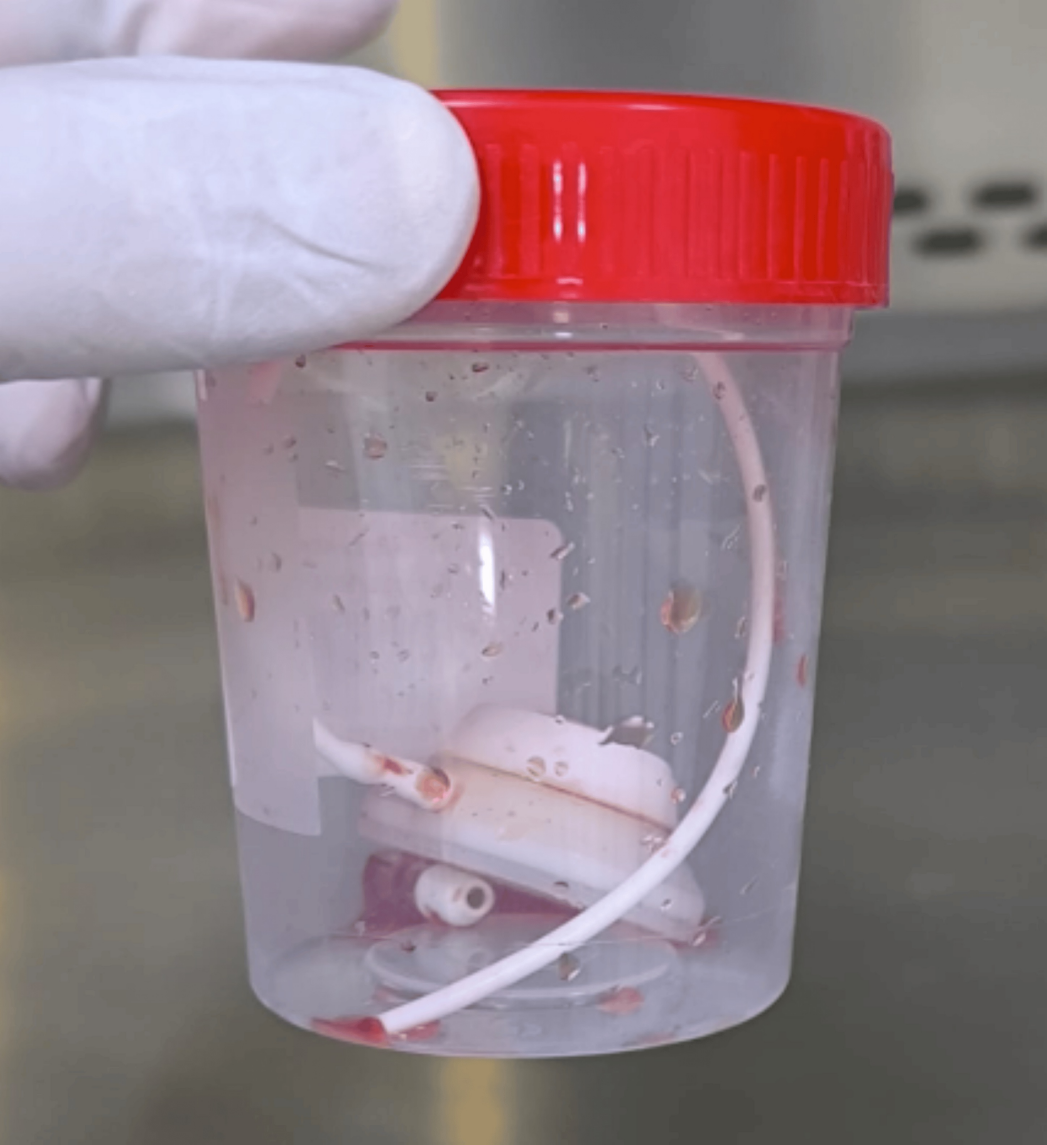
Removed implantable venous port submitted for microbiological analysis

Given the microbiological evidence supporting the port as the source of infection, source control was undertaken. The implantable venous port was extracted and submitted for microbiological analysis (Figure 5). Quantitative catheter culture was performed using the Brun-Buisson method [11] and yielded 10⁵ CFU/mL after 24 hours of aerobic incubation, confirming catheter-related infection (≥10³ CFU/mL). The isolate was identical to those recovered from blood and CSF samples.

Despite catheter removal and appropriate antimicrobial therapy, the patient’s clinical status deteriorated rapidly. He developed a profound coma and was transferred to the intensive care unit. Pancytopenia subsequently developed, likely secondary to chemotherapy and sepsis. He progressed to refractory septic shock and died within 48 hours of symptom onset despite aggressive supportive care. No sustained clinical improvement was observed despite early microbiological diagnosis, targeted antimicrobial therapy, catheter removal, and intensive supportive management.

## Discussion

This case illustrates the serious clinical course of *S. aureus* CRBSI in an immunocompromised patient with hematologic malignancy, complicated by hematogenous meningitis and rapid evolution to refractory septic shock. According to current guidelines, *S. aureus* CRBSIs are associated with a high risk of metastatic complications that require rapid diagnosis, early initiation of effective antimicrobial therapy, and immediate source control [1-3].

Patients with hematologic malignancies are particularly susceptible to invasive *S. aureus* infections due to disease-related immune dysfunction, chemotherapy-induced cytopenias, and frequent exposure to intravascular devices. In a large cohort of patients with hematologic malignancies, Venditti et al. reported that *S. aureus* bacteremia was frequently associated with central venous catheters and that severe sepsis and septic shock were linked to poorer outcomes [12]. In our case, several severity factors were present, including recent catheter insertion, underlying hematologic malignancy with chemotherapy-related immunosuppression, baseline cytopenias, and a pronounced systemic inflammatory response, reflected by rapidly rising CRP and procalcitonin levels preceding clinical deterioration.

The diagnosis of CRBSI in this case was supported by several complementary microbiological findings. The DTP indicated that blood cultures obtained from the catheter became positive more than two hours earlier than those drawn peripherally. In clinical practice, a DTP threshold of ≥2 hours is widely regarded as indicative of a catheter source when paired blood cultures are collected simultaneously with equal blood volumes [13,5]. DTP remains a valuable microbiological tool in the assessment of suspected CRBSI. Consistent with this, the Spanish Society of Clinical Microbiology and Infectious Diseases (SEIMC)/Spanish Society of Intensive Care Medicine and Coronary Units (SEMICYUC) guidelines consider a delay of at least 120 minutes between catheter and peripheral blood cultures suggestive of a catheter origin, with reported sensitivity of 72%-96% and specificity of 90%-95%; however, its interpretation depends on correct technical procedure and may be less specific in long-term catheters [5]. In accordance with these recommendations, a recent systematic review and meta-analysis demonstrated that DTP has good specificity but variable sensitivity, indicating that a positive result supports the diagnosis, whereas a negative result does not definitively exclude the diagnosis [14]. In our patient, DTP was therefore interpreted in conjunction with the quantitative catheter culture and the recovery of concordant MSSA isolates from blood, CSF, and catheter samples. This microbiological evidence was further strengthened by quantitative catheter culture using the Brun-Buisson method, which yielded 10⁵ colony-forming units per milliliter (CFU/mL) of *S. aureus*, exceeding the diagnostic threshold for catheter-related infection (≥10³ CFU/mL) [10]. The recovery of the same MSSA strain with concordant susceptibility profiles from central and peripheral blood cultures, CSF, and the catheter culture established definite CRBSI with secondary metastatic dissemination.

Hematogenous *S. aureus* meningitis is rare but represents a particularly severe manifestation of invasive staphylococcal disease. Recent studies on adult community-acquired *S. aureus* meningitis confirm that extracranial foci, most frequently endocarditis or spondylodiscitis, are commonly identified, and mortality remains high [8]. In our patient, CSF analysis demonstrated classic findings of acute bacterial meningitis, despite an initially negative Gram stain. This finding aligns with previous reports suggesting that direct CSF examination may be negative early in the disease course or after early antibiotic administration, with culture remaining the diagnostic gold standard [15].

Comparable cases documented in the literature underscore the high mortality of catheter-associated *S. aureus* meningitis. Suresh et al. described a fatal case of *S. aureus* bacteremia complicated by meningitis originating from a tunneled dialysis catheter, despite appropriate management [16]. Similarly, Huang et al. reported that adult *S. aureus* meningitis is linked to a significant mortality rate of 35.3% even with optimal management [9]. The present case is consistent with these findings and further illustrates the rapid and severe progression that can occur in immunocompromised patients.

From a therapeutic standpoint, current guidelines strongly recommend early removal of infected intravascular devices and initiation of effective systemic antimicrobial therapy in cases of *S. aureus* CRBSI [1,2]. In adults with suspected acute bacterial meningitis, empirical treatment includes vancomycin combined with a third-generation cephalosporin, followed by de-escalation to high-dose anti-staphylococcal β-lactams once MSSA is identified [4,17]. Reviews of *S. aureus* bacteremia consistently report improved outcomes with β-lactam therapy compared with glycopeptides for MSSA infections [4]. In our case, empirical therapy and subsequent de-escalation to intravenous cloxacillin were appropriate; however, despite guideline-concordant management and catheter removal, the patient progressed rapidly to refractory septic shock.

Given that *S. aureus* bacteremia carries a substantial risk of infective endocarditis and other metastatic foci, systematic evaluation is recommended. The 2023 European Society of Cardiology guidelines emphasize the importance of echocardiographic assessment in patients with *S. aureus* bacteremia, particularly when metastatic infection is suspected [18].

## Conclusions

This case illustrates the severe consequences of *S. aureus* CRBSI in immunocompromised patients. It underscores the need to promptly investigate early fever after port placement, to consider hematogenous central nervous system involvement when neurological symptoms occur, and to prioritize rapid source control when catheter-related infection is suspected. A localized catheter infection can rapidly progress to bacteremia, meningitis, and refractory septic shock, even when appropriate diagnostic and therapeutic interventions are employed. The case emphasizes the diagnostic utility of DTP and quantitative catheter culture in confirming CRBSI, and it supports current recommendations for early source control and targeted anti-staphylococcal therapy. Once severe sepsis and central nervous system involvement occur, mortality remains high, reinforcing the necessity for vigilant monitoring and prompt multidisciplinary intervention after catheter placement.
